# Pregnancy trained decidual NK cells protect pregnancies from harmful *Fusobacterium nucleatum* infection

**DOI:** 10.1371/journal.ppat.1011923

**Published:** 2024-01-12

**Authors:** Rebecca Kotzur, Shira Kahlon, Batya Isaacson, Moriya Gamliel, Yoav Charpak-Amikam, Judy Lieberman, Gilad Bachrach, Debra Goldman-Wohl, Simcha Yagel, Ofer Beharier, Ofer Mandelboim

**Affiliations:** 1 The Lautenberg Center for General and Tumor Immunology, Institute for Medical Research Israel-Canada, The Hebrew University Hadassah Medical School, Jerusalem, Israel; 2 Program in Cellular and Molecular Medicine, Boston Children’ Hospital and Department of Pediatrics, Harvard Medical School, Boston, Massachusetts, United States of America; 3 The Institute of Dental Sciences, The Hebrew University-Hadassah School of Dental Medicine, Jerusalem, Israel; 4 Department of Obstetrics and Gynecology, Hadassah-University Hospital, Mt. Scopus, Jerusalem, Israel; University of Illinois, UNITED STATES

## Abstract

Natural killer cells (NKs) found during pregnancy at the maternal-fetal interface named decidual (d)NKs, show signs of education following first pregnancy, resulting in better placentation and fetus-growth, hence termed pregnancy trained dNKs (PTdNKs). Here we show that PTdNKs provide increased protection of the fetus from *Fusobacterium nucleatum* (*FN*) infection. We demonstrate that PTdNKs secrete elevated amounts of the bacteriocidal protein granulysin (GNLY) upon incubation with *FN* compared to dNKs derived from first pregnancies, which leads to increased killing of *FN*. Furthermore, we showed mechanistically that the GNLY secretion is mediated through the interaction of the *FN*’s Fap2 protein with Gal-GalNAc present on PTdNKs. Finally, we show in vivo, using GNLY-tg mice that enhanced protection of the fetuses from FN infection is observed, as compared to wild type and that this enhance protection is NK cell dependent. Altogether, we show a new function for PTdNKs as protectors of the fetus from bacterial infection.

## Author summary

During this project, we aimed to elucidate the influence of natural killer (NK) cells in the protection of fetuses during pregnancy. At the maternal-fetal interface a special subset of NK cells can be found, the decidual NK (dNK) cells, which have been shown to be rather nurturing, in contrast to the cytotoxic peripheral blood NK cells. These cells are thought to be essential for the healthy development of placenta and fetus in the uterus. Bacterial and viral infections are quite common during pregnancy and pose a significant risk for the fetus and its development. In this manuscript, we describe how the dNK cells primed through first pregnancy show specific antibacterial properties against the bacterium *Fusobacterium nucleatum* via the lytic protein granulysin in contrast to the peripheral blood NK cells. Additionally, we showed that NK cells expressing granulysin in mice protect the developing fetuses from the effects of a maternal infection with the bacterium.

## Introduction

Natural killer (NK) cells are an important part of the innate immunity, essential in fighting infections and cancerous cells, while sparing the body’s own healthy cells [1]. The NK cell activity is tightly controlled by a complex system of activating and inhibiting receptors [2–5]. In recent years, a curious phenomenon regarding these “innate” cells has been observed, first in mice and monkeys, and later in humans, as it was demonstrated that NK cells have a certain type of memory [6–11].

NK cells are known to exist in different organs all throughout the body, specialized towards the immunological challenges specifically found at these sites [12,13]. The focus of this study are the decidua resident decidual NK cells (dNK). During the first trimester of pregnancy, 50–80% of the lymphocytes present in the decidua (the maternal uterine mucosa located directly at the maternal-fetal interface) are dNK cells characterized by being CD56^bright^, CD16^neg^ [14]. The dNK cells display a distinctly different behavior compared with the blood circulating NK cells which only contain 5–10% of this specific CD56^bright^CD16^neg^ subset [15]. The dNK cells are less cytotoxic and instead are able to regulate and support implantation and early pregnancy [16].

Combining the recent findings of adaptive-like memory and the unique function of dNK cells [17], Gamliel et al. discovered the existence of pregnancy trained decidual (PTd)NK cells [18,19]. Previous to that finding, subsequent pregnancies were found to have improved trophoblast invasion, as well as showing a higher degree of placental vascularization and angiogenesis than primary pregnancies [20]. The PTdNK cells were found in subsequent pregnancies and were characterized by a consistently increased expression of the receptors NKG2C and LILRB1, as well as increased secretion of IFNγ and VEGF*α* [18].

The PTdNK cells are hypothesized to remember first pregnancies and to enable more efficient support for succeeding pregnancies [18]. Confirming these results, a recent study characterizing the maternal-fetal interface on a single cell level revealed three different dNK cell subsets, one of them, dNK1, is shown to express the receptor LILRB1 in a higher abundance than the other two, whereas the elevated NKG2C expression is shared with dNK2 as well [21].

During pregnancy, several infections can spread from mother to child and are known to endanger the survival and development of the growing fetus. These infections are often combined under the acronym TORCH, comprising viruses, parasites and bacteria, and have been known for decades [22,23]. Despite this knowledge, these infections are still a major threat to fetuses and mothers around the globe due to the sheer variety of different pathogenic agents and transmission routes. In addition to these already well characterized and manyfold observed infections, many still unknown bacterial infections are able to induce adverse pregnancy outcomes accounting to up to 10–25% of all stillbirths [24].

The pathogenic organism of choice for this investigation is the Gram-negative anaerobic bacterium *Fusobacterium nucleatum* (*FN*). This bacterium is highly involved in periodontal infections [25], but has also been linked to adverse pregnancy outcomes despite its absence from the regular vaginal bacterial flora. This has led to the hypothesis of hematogenous transfer from the oral cavity due to gingival bleeding to the uterus followed by colonization of the decidua and placenta and subsequent spread to the fetus [24,26]. *FN* can act directly invasive and adherent, and indirectly pro-inflammatory [27]. Parhi et al. showed that *FN*’s adhesin Fap2 recognizes the trophoblasts’ Gal-GalNAc (Galβ1-3GalNAc), which expression levels are shown to rise during pregnancy, leading to binding of the bacteria to murine and human placentas [27].

Gal-GalNAc also belongs to a group of antigens presented in abundance on specific tumors [28]. But Gal-GalNAc is not the only ligand of Fap2, since the bacterium is known to bind via Fap2 to the inhibitory NK cell receptor TIGIT in order to suppress NK and T cells and therefore evade associated tumor cell killing [29]. Additionally, *FN* is able to bind CEACAM1, another inhibitory receptor, via the surface protein CbpF and to consequently inhibit immune cell activities [30].

Regarding the question how dNK cells are able to selectively kill intracellular bacteria without harming the fetus, the antimicrobial peptide granulysin (GNLY) seems to be the part of the answer. It was shown that GNLY on its own was able to annihilate intracellular bacteria inside of infected trophoblasts, without lysing the host cells through insertion into the infected cell via nanotubes [31]. All in all, these experiments showed that the presence of GNLY improved the pregnancy outcome in a murine model infected with *Listeria monocytogenes* [32].

Considering all the given information we wanted to elucidate the impact of PTdNK cells on the clearance and survival of fetuses during *FN* infection in pregnancy. We demonstrated that PTdNK cells’ increased secretion of GNLY is able to reduce the bacterial load in vitro, and that GNLY expression in murine model prevents further adverse pregnancy outcomes and weight differences in fetuses in vivo. Additionally, we demonstrated that the interaction between Fap2 and Gal-GalNAc is exclusively expressed on the dNK cells, leading to their activation.

## Results

### PTdNK cells secrete more GNLY when challenged with *FN*

**Fig 1:**
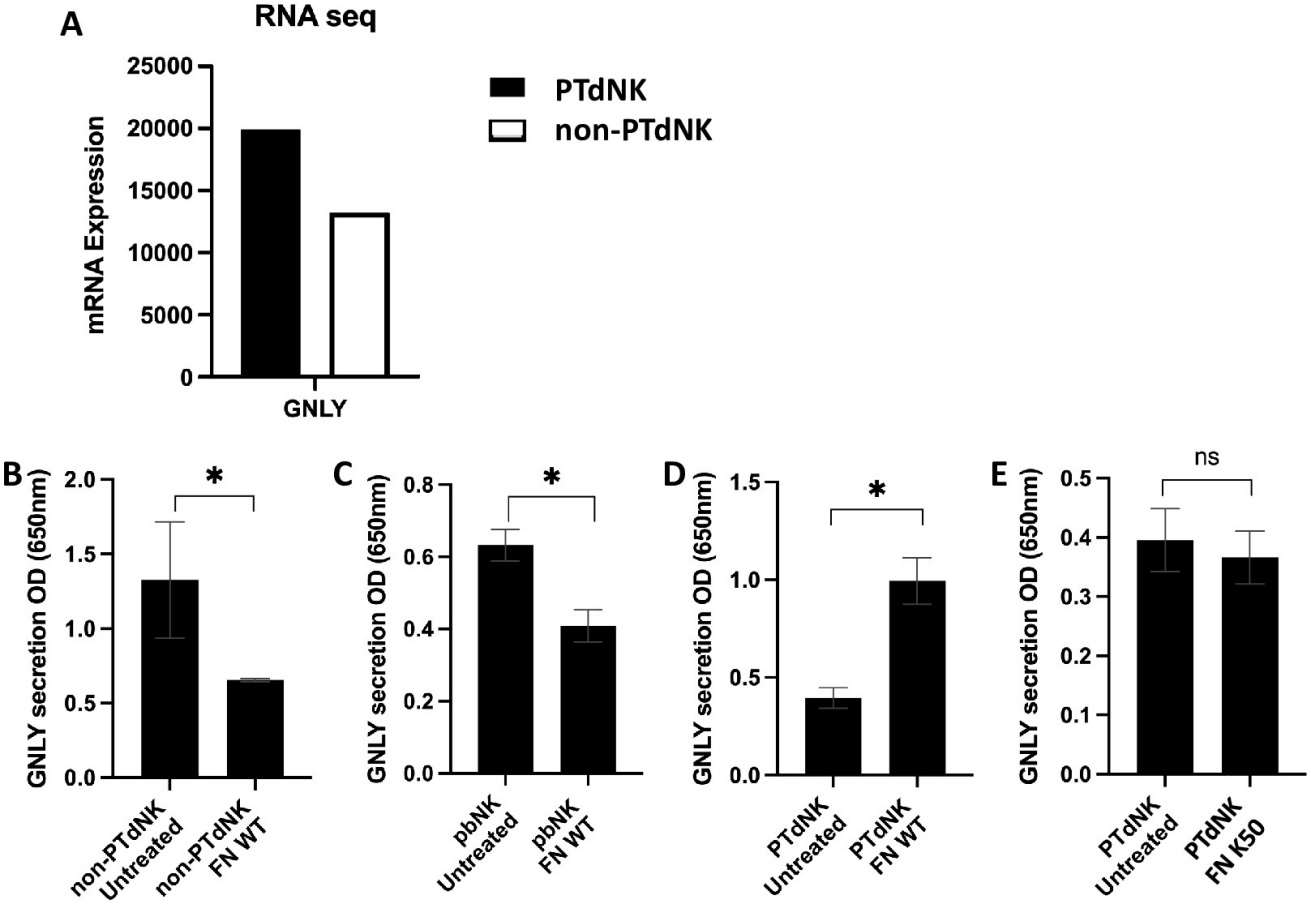
Granulysin (GNLY) expression and secretion by pregnancy trained decidual NK cells (PTdNKs) compared to non-PTdNKs. (A) mRNA Expression of the GNLY gene compared between pooled PTdNK and non-PTdNK cells samples, black = PTdNK cells, gray = non-PTdNK cells. (B) ATAC seq signal amplification of chromatin detected on chromosome 2 at position 85918877–85956340, encoding the GNLY gene, black = PTdNK cells, gray = non-PTdNK cells. (C-F) GNLY secretion detected in sandwich-ELISA measured at 650nm after incubation with (C-E) Wildtype (WT) Fusobacterium nucleatum (*FN)* and (F) Fap2 deficient *FN* mutant (*FN* Fap2 def. mut. named K50). (C) shows non-PTdNK cells’ secretion of GNLY after incubation with PBS (untreated) and WT *FN*, (D) pbNK cells’ GNLY secretion after incubation with PBS (untreated) and WT *FN*, (E) PTdNK cells’ GNLY secretion after incubation with PBS (untreated) and WT *FN* and (F) GNYL secretion of PTdNK cells following incubation with PBS (untreated) and *FN* K50. Non-PTdNK cells were obtained from first pregnancy, pb = peripheral blood NK cells. PTdNK cells were obtained from repeated pregnancies. * = p<0.05; (C-F) Depicted are 1 out of 2 representative repeats.

To investigate whether PTdNKs [18] will be able to better kill bacteria as compared to non-PTdNKs we searched for effector molecules that are expressed more on PTdNKs. One of the major differences we observed was that mRNA expression of GNLY, which was higher in pooled PTdNK samples, derived from subsequent pregnancies as compared to the pooled non-PTdNK samples (Fig 1A).

Next, we investigated whether the interaction of PTdNKs with *Fusobacterium nucleatum (FN*) will result in GNLY secretion. We compared GNLY secretion from non-PTdNKs derived from first pregnancies, peripheral blood (pb)NKs and PTdNKs after 72h of co-incubation. As can be seen, incubation of non-PTdNKs and pbNKs with *FN* resulted in reduced GNLY secretion compared to the untreated control (Fig 1B and 1C, respectively). The reason for that reduction is currently unknown. In marked contrast, when PTdNKs were incubated with *FN*, a significant elevation of GNLY secretion was observed (Fig 1D). This elevation of GNLY secretion was abolished when a Fap2-deficient *FN* mutant called K50 [33] was incubated with PTdNKs (Fig 1E). Thus, PTdNK, but not non-PTdNK or pbNK cells secrete GNLY upon interaction with *FN* in a Fap2-dependent manner.

### *FN* is killed efficiently by PTdNK cells in a degranulation independent mechanism

**Fig 2:**
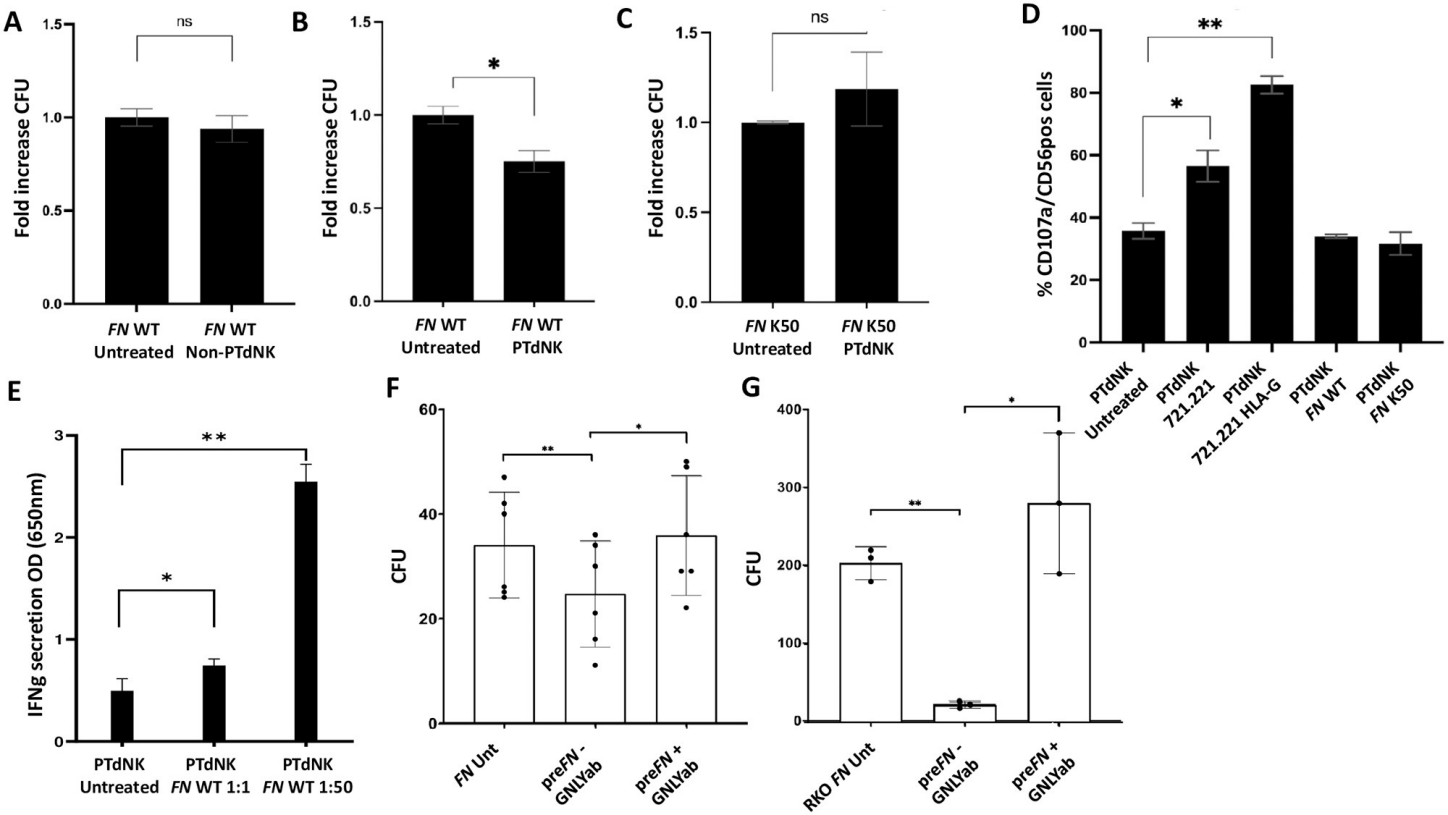
Bacteriocidal and degranulation potential of PTdNKs when encountering *Fusobacterium nucleatum* (*FN*). A-C) Bacteriocidal assay performed with A+B) Wildtype (WT) *FN*, and C) Fap2 deficient *FN* mutant (*FN* Fap2 def. mut. K50), after incubation with A) non-PTdNK cells derived from first pregnancy, and B+C) PTdNK cells, results measured in Colony forming units (CFU) compared and normalized to untreated control of *FN* only; * = p<0.05; D) Degranulation assay performed on PTdNKs incubated with 721.221 and 721.221 cells overexpressing HLA-G in their surface, as well as FN WT and FN Fap2 def. mut. K50, degranulation measured in %CD107a/CD56 positive cells after incubation detected via FACS, * = p<0.05, ** = p<0.01; Depicted are 1 out of 2 representative repeats; E) IFNγ secretion after incubation of PTdNKs with *FN* WT. Depicted is 1 out of 2 representative repeats; * = p<0.05, ** = p<0.01.; F) Bacteriocidal assay with WT *FN* utilizing supernatant gathered from PTdNK cells preincubated with WT *FN* supplemented with (pre*FN* + GNLYab) or without (pre*FN*—GNLYab) GNLY blocking antibody, depicted are 1 out of 3 representative experiments, N = 6, * = p<0.05, ** = p<0.01.G) Bacteriocidal assay with RKO cell intracellularly infected with WT *FN* utilizing supernatant gathered from PTdNK cells preincubated with WT *FN* supplemented with (pre*FN* + GNLYab) or without (pre*FN*—GNLYab) GNLY blocking antibody, depicted are 1 out of 3 representative experiments, N = 3, * = p<0.05, ** = p<0.01.

To further assess the functional abilities of PTdNKs to fight bacterial infection, we established a bacteriocidal assay directed against *FN*. During this assay, we incubated non-PTdNKs and PTdNKs with a constant amount of *FN* for 1.5h, lysed the NK cells using water, and plated the surviving *FN* for 48-72h. We then compared the colony forming units (CFU) pf untreated *FN* with that of *FN* treated with the various NK cells to determine an increase or decrease. In Fig 2 we clearly see that *FN* incubation with non-PTdNKs did not lead to a decrease in CFU (Fig 2A), but when PTdNKs were used, a significant decrease of *FN* CFU was observed (Fig 2B). Furthermore, when employing the Fap2-deficient *FN* mutant K50, the bacteriocidal effects of the PTdNKs were abolished (Fig 2C).

To investigate the mechanisms through which PTdNKs kill *FN* we used the widely established degranulation assay, measuring the percentage of CD107a on NK cell surface after incubation with target cells. We were able to see that the PTdNKs degranulate when incubated with 721.221 and that degranulation is further elevated when 721.221 HLA-G transfectant are used (probably because of the interaction of LILRB1 with HLA-G, [18]). In contrast, after incubation with *FN* (both WT and the Fap2-deficient mutant K50), no increase degranulation was observed (Fig 2D). Thus, PTdNKs kill *FN* in a Fap2-mediated, degranulation-independent mechanism. Furthermore, we investigated the change in IFNγ secretion after incubation of PTdNKs with *FN* WT, observing a significant increase indicating an INFγ-secretion mechanism independent of degranulation (Fig 2E).

We next wanted to demonstrate that GNLY is responsible for the *FN* killing. for that we preincubated PTdNK cells with WT *FN* for 72h in the presence or absence of anti-GNLY antibody. Next, we collected the supernatants of the cultured cells and incubated it with newly added *FN*, for 1.5h and determined the CFU, as described above. After incubation with WT *FN*, but only without prior addition of GNLY blocking antibody (Fig 2F). As can be seen, reduced *FN* CFU was observed following incubation of *FN* with the supernatants and this CFU reduction was completely abolished when the anti-GNLY antibody was present in the assay (Fig 2F). We next employed the same supernatants used in Fig 2F, on cells infected with *FN*. We incubated RKO cells with WT *FN* at an MOI of 50:1 for 2h for intracellular infection, selectively extinguished the remaining extracellular bacterial cells via gentamycin treatment (which cannot permeate into the cells, before treating the cells with the supernatant) and confirmed that *FN* was no longer present outside the RKO cells. The infected cells were lysed using sterile UPW to extract the bacteria before quantitative cultivation. As can be seen in Fig 2G the number of colony forming units was significantly reduced only after treatment with the supernatant that were taken from NK cells incubated with *FN* only. Importantly, in the presence of anti-GNLY antibody, a complete abolishment of the CFU reduction was observed (Fig 2G). These combined results indicate that GNLY secreted from PTdNKs is essential for the killing of extra- and intracellular *FN*.

### *FN* binds to PTdNK cells via Gal-GalNAc and not TIGIT

**Fig 3:**
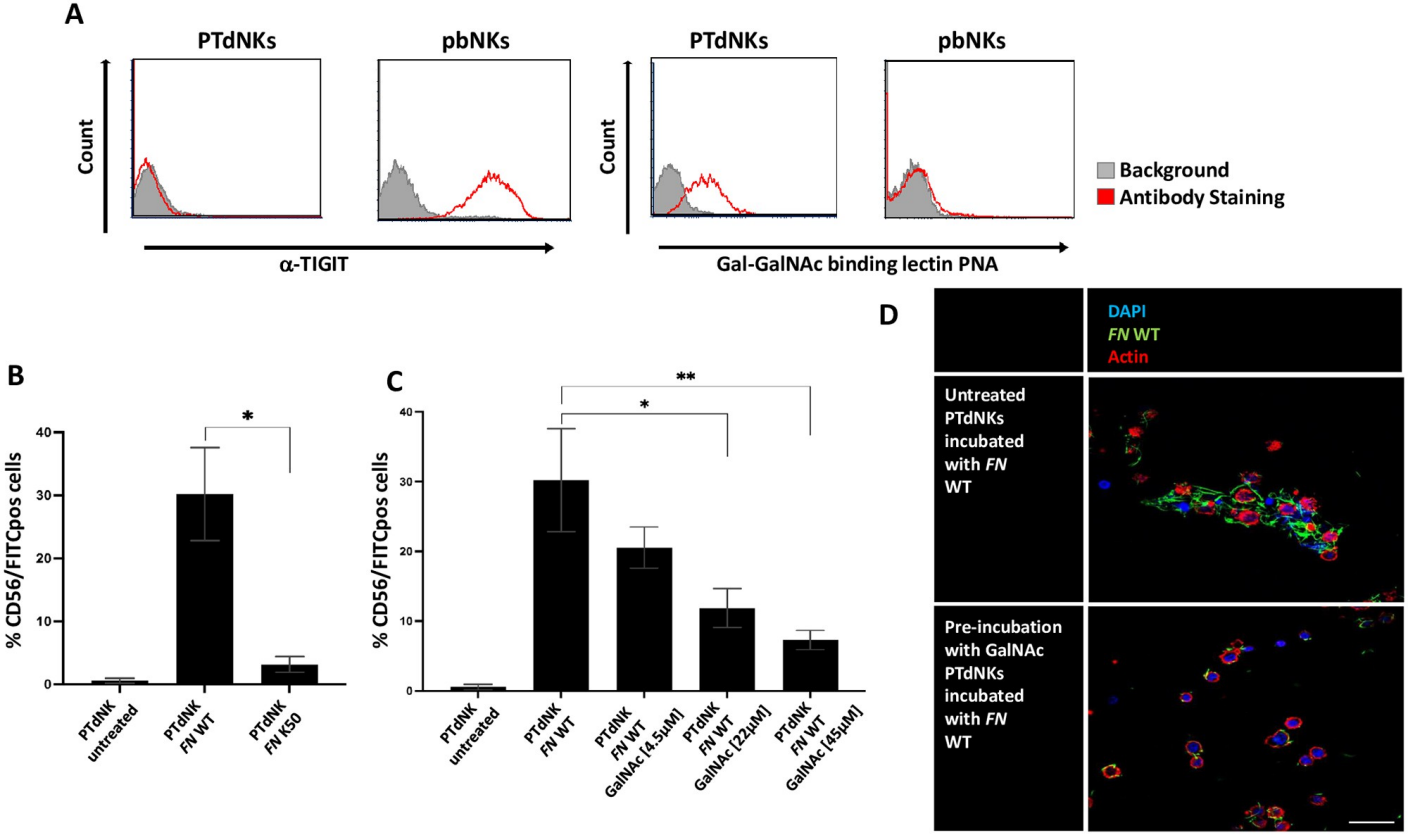
Binding of Fap2 interaction partners and FN by PTdNKs. A) Expression of known Fap2 interaction partners TIGIT and Gal-GalNAc on the surface of PTdNK and peripheral blood (pb)NK cells, TIGIT detected via anti-TIGIT antibody and Gal-GalNAc detected by Gal-GalNAc-binding lectin peanut agglutin (PNA), Gray = Background staining, Red = antibody binding; B) Binding of FN to PTdNKs, measured in %CD56/FITC positive cells, comparing untreated PTdNKs and PTdNKs incubated with *FN* WT and *FN* Fap2 def. mut., depicted are 1 out of 3 representative experiments, * = p<0.05; C) Inhibition of binding of *FN* WT to PTdNK cells by preincubation with GalNAc 4.5μM, 22μM and 45μM, measured in %CD56/FITC positive cells compared to untreated control and PTdNK cells incubated with FN without pretreatment, * = p<0.05, ** = p<0.01, depicted are 1 out of 2 representative repeats; D) Immunofluorescence staining of *FN* WT interaction with PTdNK cells with (right) and without (left) preincubation with GalNAc, magnification 60x, scale bar 60μm, Blue = DAPI, Green = *FN* WT, Red = Actin.

In the above figures we showed that the killing of *FN* by PTdNKs is Fap2-dependent. Fap2 interacts with two ligands: TIGIT and Gal-GalNAc [28,29]. To test which of these ligands is expressed on PTdNKs we detected Gal-GalNAc using the Gal-GalNAc binding lectin PNA, and for TIGIT we used an anti-TIGIT antibody. We compared the expression of both surface molecules between PTdNKs and pbNKs. In Fig 3A it can be appreciated that only the pbNK cells express TIGIT on their surface, but the PTdNKs do not. In contrast, pbNKs did not express a noticeable amount of Gal-GalNAc, whereas the PTdNKs show a high abundance of this carbohydrate. From these findings we can conclude Gal-GalNAc expressed on PTdNKs is the probable Fap2 ligand. To further corroborate these results, we labeled the WT and the Fap2-deficient *FN* mutant with FITC and determined binding to PTdNKs. In Fig 3B it can be seen that the WT *FN* efficiently binds to PTdNK cells, whereas only limited binding was observed with the Fap2 deficient mutant. To demonstrate that this binding is indeed Gal-GalNAc dependent, soluble GalNAc was preincubated with the labeled bacteria before the co-incubation with the PTdNKs. In Fig 3C a dose-dependent decrease in WT *FN* binding can be seen. Lastly, we also visualized the interaction using immunofluorescence (Fig 3D). In this figure, we can appreciate the clustering of PTdNK (blue and red) cells in the presence of *FN* (green) together generating an agglomeration of cells and bacteria. The observed accumulation is disrupted due to the use of GalNAc that was pre-incubated with *FN* before exposure to PTdNK cells (Fig 3D).

### GNLY-tg mice protect fetuses better from *FN* infection than WT mice

**Fig 4:**
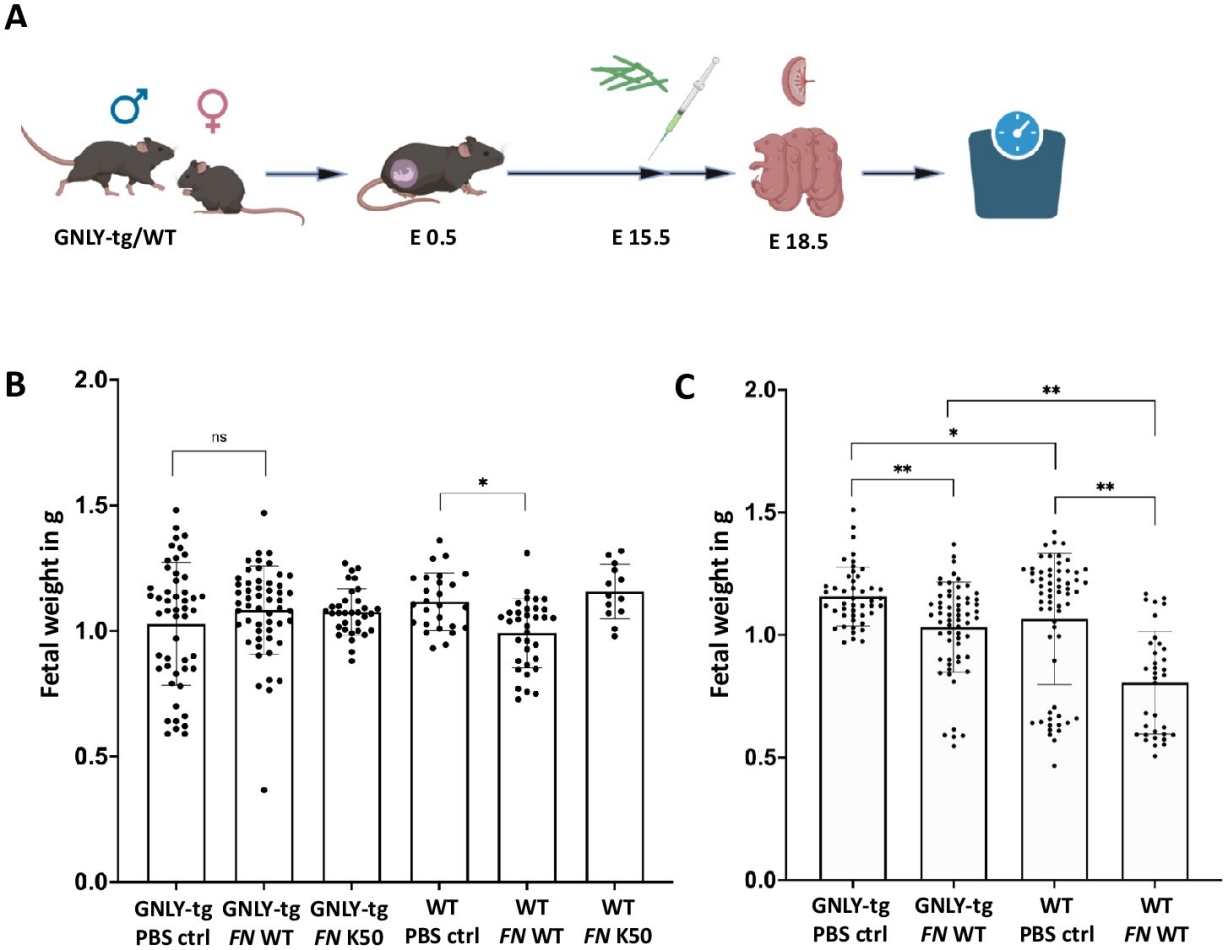
Murine GNLY-tg pregnancy model of *FN* infection in vivo. (A) Experimental scheme: GNLY-tg and Wildtype (WT) mice are coupled and day E0.5 is determined via vaginal plug discovery, establishing mating success. At day E15.5 *FN* or PBS is injected intravenously (i.v.). After 72h, the maternal mice are sacrificed, and fetuses were excised and weighted. Created with BioRender.com. (B) Weight of the fetuses derived from mice used in this study comparing the fetus weight between GNLY-tg and WT mice, PBS-, *FN* WT-, and *FN* Fap2 def. mut. K50 injected. Weight is given in grams each dot equals one fetus, n>15, (C) Weight of the fetuses derived from mice used in this study comparing the fetus weight between GNLY-tg and WT mice, PBS- and *FN* WT- injected after depletion of NK cells one day prior to injection via an anti-NK1.1 antibody. Weight is given in grams. Each dot equals one fetus, n>13, * = p<0.05, ** = p<0.01.

To assess the effect of *FN* and GNLY in vivo, we utilized humanized GNLY-tg mice [34] and a pregnancy model that was employed previously [27]. In Fig 4A a schematic experimental plan is shown. First, male and female mice were mated, and pregnancy was confirmed via vaginal mucus plug and weight gain. At day E 15.5, the pregnant mice were injected intravenously with *FN* or PBS, and after 72h the mice were sacrificed. After sacrificing the mice, the fetuses were weighted. The measured weights are depicted in Fig 4B. The fetal weight showed only a significant decrease in the WT mice infected with WT *FN*, whereas the GNLY-tg mice did not show any diminution of fetal weight compared to the PBS control (Fig 4B). This reduction in weight loss was Fap2 dependent as no reduction was observed when WT or GNLY-tg mice were infected with the Fap2-deficient mutant K50 (Fig 4B). Additionally, we characterized the immune cells’ subsets present in the mice without infection and with FN WT infection (S1A Fig). We see only a slight increase for NCR1^+^ NK cells in the GNLY-tg mice infected with FN WT, whereas the WT mice infected with FN presented an decreased NK cell amount after infection. Regarding NCR1^+^/CD3^+^ NKT cells, both mouse lines showed an increased population after infection (S1B Fig). Observing T cells in the mice after infection, we were able to discover the opposite picture. Here we see decreased CD3^+^ cells in infected GNLY-tg mice and an increased population in the WT mice. The T cells observed in these mice appeared to belong to the CD4^+^ Th cells (S1C Fig).

To demonstrate that the NK cells at the maternal-fetal interface are responsible for this effect, we depleted NK cells using anti-NK1.1 antibody in the pregnant mice one day prior to *FN* injection. The fetuses that were retained and brought to the E18.5 mark, bear a significantly lower weight compared to the fetuses in the PBS injected control mice, both in WT and GNLY-tg mice (Fig 4C), indicating that NK cells and GNLY play a significant role in mice protection from *FN* infection.

## Discussion

Here we show that PTdNK cells secrete high levels of GNLY when incubated with *FN* and possess a higher bacteriolytic activity than first pregnancy dNK or pbNK cells. We also prove that the bacteriolytic abilities of PTdNKs are mediated by the GNLY secretion upon interaction with WT *FN*. It was demonstrated by the Lieberman group that dNK cells kill intracellular pathogens found inside trophoblasts in a process which is independent of degranulation [31]. Here, we show that the GNLY-mediated direct killing of *FN* by PTdNKs is also degranulation independent and that PTdNKs can kill intracellular as well as extracellular *FN*. The exact mechanism of killing will be investigated in the future, but we established that the bacteriocidal abilities of the PTdNKs rely on the presence and secretion of GNLY upon activation of the cells. We demonstrate that *FN* interacts with PTdNK via the bacterial virulence factor Fap2 and the carbohydrate moiety Gal-GalNAc present on PTdNKs but not on pbNK. Indeed, when the Fap2-deficient *FN* mutant strain, K50, was used a complete abolishment of the bacteriolytic, secretory and even general binding capacity to the PTdNK cells was observed. The same effect was observed when *FN* WT was incubated with GalNAc before interacting with the NK cells. The expression of Gal-GalNAc on PTdNKs and not on pbNKs is an interesting matter and has not been extensively investigated yet. There are two alternatives that will be investigated in the future: one is elevated levels of GalNAc synthesis in PTdNKs and the other is a removal of terminal sialic acids residues.

Our murine in vivo pregnancy model uncovered that transgene human-GNLY expressing mice are not able to prevent infection with *FN* of the fetuses, decidua and placenta. Nevertheless, we observed no significant differences in the weight of the fetuses undergoing this infection. In contrast, reduced fetus weights were observed when WT mice that do not express GNLY were used, suggesting that GNLY reduces the infection’s effect on the fetuses. When we depleted NK cells in the GNLY tg mice before injection with *FN* WT, this effect was abolished, and we were able to see a significant weight loss in the fetuses derived from infected pregnant mice. Thus, GNLY in NK cells is responsible for the observed effects. Nevertheless, we still observed a protective effect in the mice. This is congruent with the findings in S1 Fig showing an increase of T cells in the mice after infection. The maternal-fetal interface is comprised of a high percentage of immune cells, apart from NK cells, which can influence the infection of the fetus and the severity of the infection’s effects [35]. The cells’ composition changes during pregnancy progression, from a predominantly NK cell population in the first trimester, towards a more T cell dependent mixture. The T cells found at the maternal-fetal interface are only in their early stage of being further characterized and are already known to comprise a complex mixture of Th1, Th2, Th17 and Treg cells [35, 36]. GNLY is not only expressed by NK cells, but also by cytotoxic lymphocytes, explaining the remaining effect at the maternal-fetal interface in the NK cell-depleted mice [37].

To put this research into scientific context of NK cell education in pregnancy, we can say that there are definite indices for education of the dNK cells during first pregnancy regarding their reactivity towards pathogenic threats. As mentioned before, the cell population first described in Gamliel et al. termed PTdNK showed a higher abundance of NKG2C and LILRB1, and higher availability of genes involved in secretion of inflammatory molecules [18]. This dNK cells subset was further characterize by the Teichman group while mapping the maternal-fetal interface on the single cell level. Their DNK1 cell population strongly resembles the PTdNK cells [21]. Combining these recent findings with the work from the Lieberman group regarding the involvement of GNLY in the prevention of adverse pregnancy outcomes after infection with *Listeria monocytogenes* [31], we can position our research in the realm of further investigating and characterizing the mechanisms at play during dNK cell education. So far, the exact mechanism of education, as well as the precise pathway involved in activating the enhanced bacteriolytic properties of the trained dNK cells, have not been unveiled. Showing a concrete way of interaction between the pathogen and the dNK cells poses a major new alleyway for further investigation and characterization of downstream pathways involved in this activation.

To sum up the presented work, we can say that dNK cells get educated and trained during the first pregnancy and are functionally able to protect the fetus better from the harmful effects of maternal infection with *FN* during pregnancy. The findings can potentially be translated into a more clinical and cancer biologically frame since the association between *FN* and tumorigenesis are becoming more and more evident and researched [27,38,39]. Modifications of the PTdNK cells helping to fight the bacteria can be proven to be beneficial in other settings as well and combined with modern immunotherapy like CAR-NK cell these results of training can be used to disrupt the tumor microenvironment containing *FN* leading to a better outcome of immunotherapy.

### STAR Methods

**Table:** 

REAGENT or RESOURCE	SOURCE	IDENTIFIER
Antibodies		
Purified anti-human Granulysin	Biolegend	BLG-348008
Biotin anti-human Granulysin	Biolegend	BLG-526104
Peroxidase-conjugated Streptavidin	Jackson ImmunoResearch	016-030-084
Anti human CD56 APC	Biolegend	BLG-318310
Anti human CD56-PE	BD Biosciences	MAB-345810
Anti human CD107a-APC	Biolegend	BLG-328620
FITC anti hLILRB1	R&D	FAB-20171F
InVivoMAb anti mouse NK1.1, clone PK136	Bio X Cell	BE0036-25
Purified anti-human IFNγ	Biolegend	BLG 502402
Biotin anti-human IFNγ	Biolegend	BLG 502504
Anti mouse NCR1-PE	Biolegend	BLG 137604
Anti mouse CD3-APC	Biolegend	BLG 100312
Anti mouse CD4-PE	Biolegend	BLG 100408
Anti mouse CD8a-PE	Biolegend	BLG 155008
Bacterial and virus strains		
*Fusobacterium nucleatum* WT ATCC 23726	ATCC	23726
*Fusobacterium nucleatum* K50	Bachrach Lab	
Chemicals, peptides, and recombinant proteins		
Fluorescein isothiocyanate (FITC)	Sigma-Aldrich	500MG-F7250
GalNAc	Sigma-Aldrich	A2795; CAS 1811-31-0
PFA paraform aldehyde	bar naor	BN15710
CAS-Block Histochemical Reagent	ThermoFisher Scientific	008120
DNase type I	Roche	10104159001
Collagenase IV	Worthington	LS004186
rhIL-15	BLG	570302
Rhodamine Phalloidine	ThermoFisher Scientific	R415
DAPI Solution (1 mg/mL) / 1 mL	Sigma-Aldrich	TS-62248
Critical commercial assays		
EasySep Human NK Cell Enrichment kit	StemCELLS	19055
Deposited data		
Transcriptome analysis comparing NKG2C^hi^ and NKG2C^neg^ cells	GEO database	GSE79879
Experimental models: Cell lines		
721.221 cells	In house	
721.221 HLA-G transfectants	In house	
Experimental models: Organisms/strains		
GNLY-Tg C57BL/6	Lieberman Lab	
C57BL/6	Invigo	
Oligonucleotides		
AGAGTTACCCAGGGCCTCGT	Merck	GNLY fwd
AGAGGCACATCCTGGAAGC	Merck	GNLY rev

### Ethics statement

This study was carried out in strict accordance with the recommendations in the guide for the care and use of laboratory animals and the protocol was approved by the Ethics Committee of the Hebrew University of Jerusalem under the approval number: MD-20-16096-3. All efforts were made to reduce the number of animals, refine their living conditions and minimize their suffering.

For human samples the institutional review board of the Hadassah Hospital Medical Center approved the use of decidual material, according to the principles of the Helsinki Declaration (0423-10-HMO). All women were healthy, and pregnancies were terminated due to social reasons. Written informed consent was obtained from the women.

### Mice

GNLY-Tg C57BL/6 were described previously [32,34]. Mice were bred and maintained in specific-pathogen free (SPF) conditions within the animal facility of the Hebrew University, Jerusalem until infection with *Fusobacterium nucleatum*. The experiment was approved by the Ethics Committee of the Hebrew University of Jerusalem under the approval number: MD-20-16096-3. Virgin female mice (6–8 weeks old) were mated with male mice and the appearance of a vaginal plug and weight gain was used to mark E 0.5 and to establish maintenance of pregnancy. Pregnant animals were infected on E 15.5. All pregnant animals were included in the final analysis.

### Bacteria

*FN* strains (WT ATCC 23726 and Fap2-deficient mutant K50) were grown on anaerobic blood agar supplemented with 5% defibrinated sheep blood (Novamed) under anaerobic conditions generated using Oxoid AnaeroGen 2.5L Jars and Sachets (Thermo Fisher).

### dNK cells preparation

Preparation of decidual sample was previously described in Gamliel et al. 2018 [18] and were obtained from healthy women who underwent elective first trimester terminations of normal pregnancies. The institutional review board of the Hadassah Hospital Medical Center approved the use of decidual material, according to the principles of the Helsinki Declaration (0423-10-HMO) and informed consent was obtained from all women. All women were healthy, and pregnancies were terminated due to social reasons. Written consent was obtained from the women. A fetal ultrasound was performed prior to all elective terminations to establish the fetal age by means of CRL (crown-rump length) and fetal viability. Decidual tissues were rinsed of blood clots and trimmed to 1mm pieces in 1xPBS supplemented with 1mM of penicillin- streptomycin. Tissue pieces were then transferred to a 50 ml tube with 15 ml warm RPMI-1640 and subjected to enzymatic digestion with 0.1 mg per 1 ml DNase type I (Roche) and 1 mg per 1 ml Collagenase IV (Warthington). Digestion was performed for 20 min in a 37°C water bath with vigorous shaking every 5 min. After collection of the supernatant, the remaining tissue pieces were treated with two additional rounds of digestion. The supernatants obtained from three digestion rounds were passed through a 40-mm cell strainer. Cells were then subjected to sterile density gradient separation by Ficoll and collected at the interface.

### Bacteriocidal assay

dNK cells were isolated and prepared as described above and maintained overnight in the IL-15 supplemented NK medium without additions of antibiotics. *FN* was prepared as described above and distributed in 2.5x10^3^ CFU per 100μl per well in antibiotic-deprived NK medium. The activated dNK cells were added in various ratios per 100μl per well to the bacteria. This suspension was incubated for 1.5h at 37°C and 700μl UPW were added to dissolve effector cells. The suspension was dropped on anaerobic blood agar plates in triplicates and incubated for 72h at 37°C under anaerobic conditions generated using Oxoid AnaeroGen 2.5L Jars and Sachets (Thermo Fisher). Grown and visible CFUs were counted and normalized to the untreated bacterial control.

For the intracellular infection model, 1x10^5^ RKO cells were incubated with WT *FN* at MOI 50:1 for 2h at 37°C in DMEM medium without antibiotics. After the infection and washes, the infected cells were incubated with medium containing Gentamicin 50 μg/ml for 1h to kill remaining extracellular bacterial cells. Following this, the cells were incubated with the supernatant gathered from the incubation of PTdNK cells with *FN* supplemented with and without 5μg GNLY-blocking antibody (Biolegend) for 2h at 37°C. The RKO cells were lysed with sterile UPW and the surviving intracellular bacteria were plated and incubated as described before.

### Secretion assay

dNK cells were isolated and prepared as described above and 1 x 10^5^ dNK cells were incubated with various ratios of *FN* K50 lacking Fap2 [33] and WT strains; 1:1, 1:50, 1:100 and 1:500, for at least 72 h in DMEM medium containing 10% human serum and rhIL-15 (Biolegend) (0.1μ/ml). The resulting supernatants were used for an ELISA against GNLY and IFNg after centrifugation. For assessment of GNLY dependency on the bacteriocidal effect of PTdNKs, additional secretion assays were performed in a similar fashion utilizing the incubation in a ration 1:50 while supplementing 5μg of GNLY-blocking antibody (Biolegend) for 72 h at 37°C.

### ELISA

High absorbent ELISA 96 flat plates were precoated with purified anti granulysin and anti IFNγ antibody (Biolegend) 1ug/ml in 50 ul PBS x 1 for 2h at 37°C and blocked by 200 ul of blocking buffer (1% BSA in PBS x 1) for 2h at room temperature. Following 3 times washing with PBS x 1/tween, 100μl of the supernatant from the secretion assay were added and incubated over night at 4°C. After discarding the supernatant and 4 times wash with PBS x 1/tween, the plate was incubated with biotinylated antibody against granulysin and IFNg (Biolegend) 1 ul/ml in 100 ul PBS x 1/tween + 1% BSA for 1h at room temperature. Incubation with Streptavidin HRP (Jackson ImmunoResearch) 1 ul/ml in PBS x 1/tween + 1% BSA for 30min at room temperature was preceded by and followed by wash with PBS x 1/tween, before ultimate incubation with 100μl TMB solution immediately before measuring the fluorescence in the plate reader.

### Bacterial binding/FACS stainings/blocking

*FN* (1x10^9^ CFU/ml) was labeled with fluorescein isothiocyanate (FITC) (Sigma-Aldrich) (0.1 mg/ml in PBS; Sigma-Aldrich) for 30 min at room temperature and washed three times in PBS. FITC labeled bacteria were incubated with dNK cells at various bacteria to cell ratios for 2h at 37°C. Additionally, *FN* was preincubated with various amounts of GalNAc (Sigma-Aldrich) after labeling, but before incubation with dNK cells. Cells were washed and bacterium binding was detected using flow cytometry.

For immune cells’ characterization in maternal mice, the cells were harvested from the spleen and washed with PBS before staining. For the staining 5x10^4^ cells were resuspended in FACS medium and incubated with various antibodies (NCR1-PE, CD3-APC, CD4-PE and CD8a-PE (Biolegend)) for 30 min at 4°C. After the staining, the cells were washed twice and resuspended in FACS medium for reading. Reading was done in the Cytoflex (Beckman & Coulter) and analysis was performed via FCS Express 6/7.

### Immunofluorescence

*FN* was grown and labeled with FITC as described above. After labeling, 1x10^6^ CFU of the bacteria were fixated onto an 8-chamber slide (Bar-Naor) via 4%PFA over night at 4°C. dNK cells were prepared as described above and after permeabilization of the fixated bacteria, co-incubated on the chamber slide for 2h at 37°C. After co-incubation, the slides were washed with ice-cold 1xPBS (3000 G, 4°C, 5 min) and then blocked using CAS-Block (ThermoFisher Scientific) for 1.5h at 4°C. The cells were washed again in 1xPBS 3 times and then incubated with various antibodies for 2h at 4°C: APC-CD56 (Biolegend); Rhodamine Phalloidine (ThermoFisher Scientific). Then the cells were washed twice in ice-cold 1xPBS. The cells were incubated in the presence of DAPI (Sigma-Aldrich) (0.1 mg/ml, diluted in 1xPBS) for 25 min, washed twice in ice-cold 1xPBS and fixated using 4% PFA (Bar-Naor Ltd) overnight. Finally, the cells were mounted on an 8-chamber slide (Bar-Naor) and visualized using an Olympus Fluoview FV1000 confocal microscope.

### Degranulation assay

dNK cells were prepared as described above and incubated with 721.221 cells, 721.221 HLA-G transfectants in a 1:1 ratio, and with *FN*, both WT and K50 mutant in a 1:50 ratio for 2h at 37°C. dNK degranulation was assessed by FACS using CD56-PE (0.5μl/100μl, BD Biosciences) and CD107a-APC (0.5μl/100μl, Biolegend). For analysis of degranulation, NK cells were gated according to their appearance in the forward/side scatter and for CD56 expression. CD107a positive percentage was determined on the gated NK cell population.

### Pregnancy model

C57BL/6 (both WT and GNLY-tg) mice were coupled, and mating was determined by the presence of a white vaginal plug and consecutive weight gain. The day when the plug was detected was termed E0.5 of gestation. The pregnant mice were infected by *FN* ATCC 23726 wild type (WT) and the *FN* Fap2 mutant K50 by tail vein injection as follows. An aliquot of 100 mL of the bacterial suspension (5x10^7^ CFU) or sterile PBS was injected into the tail vein. After 72 hours, the placentas, fetuses, deciduas and maternal spleens were harvested from each pregnant mouse and homogenized under sterile conditions.

NK cells were depleted one day before *FN* injection via i.v. injection of 25μg InVivoMAb anti mouse NK1.1, clone PK136 (Bio X Cell). The injection was applied i.v. due to the progressed pregnancy of the mice.

### RNAseq data

Detailed results of the Transcriptome analysis comparing NKG2C^hi^ and NKG2C^neg^ cells from multigravid dNK cells is accessible in the GEO database, under accession GSE79879 [18].

### Statistical analysis

Statistical analysis was performed by pairwise two-sided Student’s T-test and one-sided Mann-Whitney U test, significance indicated in the figure legends.

## Supporting information

S1 Fig Characterization of immune cells in the maternal mouse before and after infection with *FN*. (A) Histograms describing the maternal spleenocytes of GNLY-tg and WT mice, injected with PBS (not infected) and *FN*. Depicted are double positive stainings with NCR1, CD3; CD4, CD3 and CD8, CD3. The arrows indicate the antibodies. 1 out of 3 representative repeats shown. B) Graph quantifying the percent of positive cells for NCR1 and NCR1/CD3; * = p<0.05, ** = p<0.01. C) Graph quantifying CD3, CD4, CD4/CD3, CD8 and CD8/CD3 positive cells; * = p<0.05, ** = p<0.01.
(TIF)

S1 Data Raw data. (XLSX)
