# Supplementary figures and images for: Pregnancy trained decidual NK cells protect pregnancies from harmful Fusobacterium nucleatum infection
Source: PLoS Pathog. 2024 Jan 12;20(1):e1011923. doi: 10.1371/journal.ppat.1011923 (PMC10826933; doi:10.1371/journal.ppat.1011923)

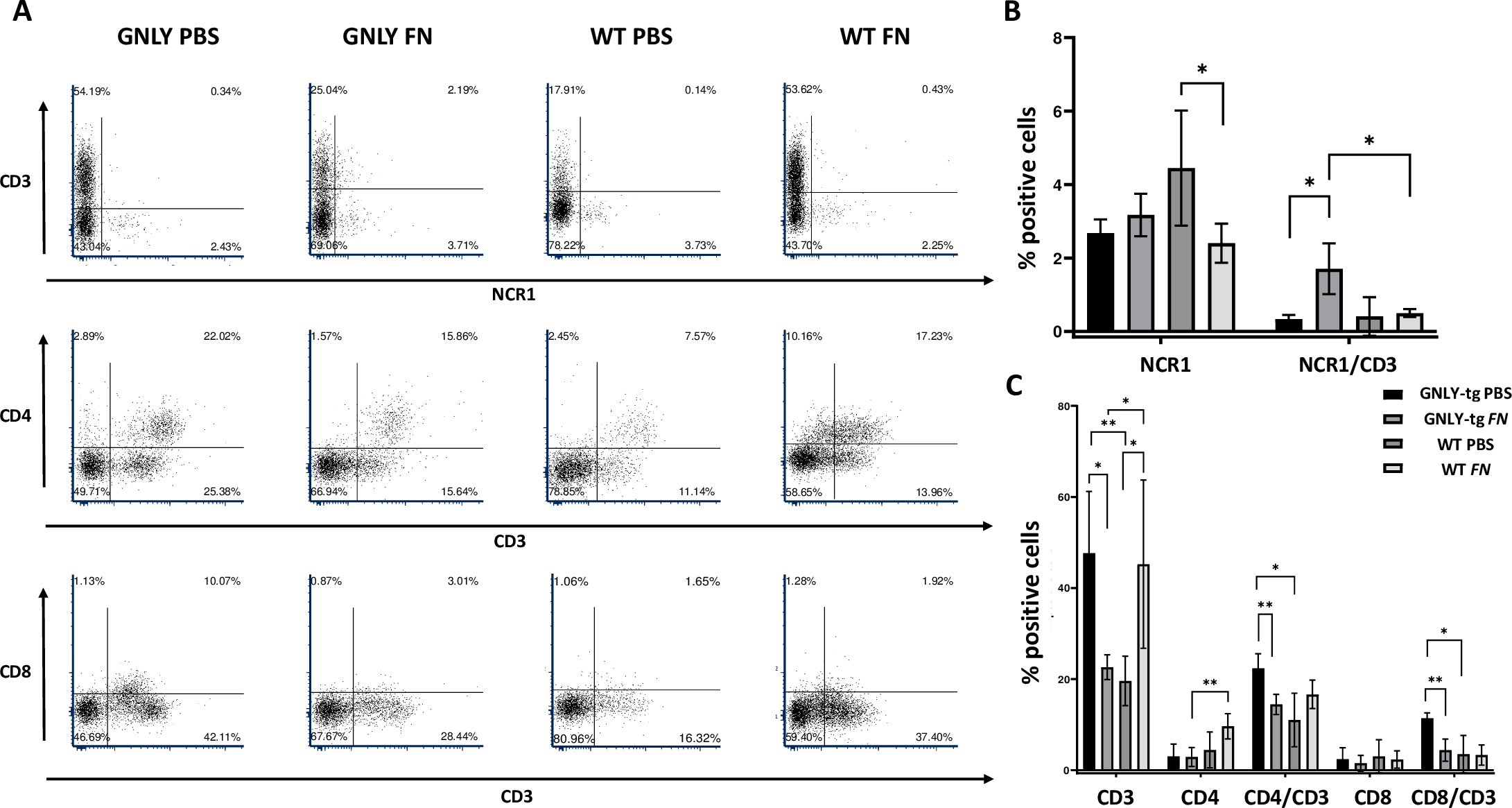

Supplement: S1 Fig — (A) Histograms describing the maternal spleenocytes of GNLY-tg and WT mice, injected with PBS (not infected) and FN. Depicted are double positive stainings with NCR1, CD3; CD4, CD3 and CD8, CD3. The arrows indicate the antibodies. 1 out of 3 representative repeats shown. B) Graph quantifying the percent of positive cells for NCR1 and NCR1/CD3; * = p<0.05, ** = p<0.01. C) Graph quantifying CD3, CD4, CD4/CD3, CD8 and CD8/CD3 positive cells; * = p<0.05, ** = p<0.01. (TIF) [file ppat.1011923.s001.tif]
